# Evaluating the Combined Effects of Elevated [CO_2_] and Accelerated NPQ Relaxation on Yield, Physiology and Transcription in Soybean

**DOI:** 10.1111/pce.70608

**Published:** 2026-05-11

**Authors:** Dilkaran Singh, Amanda P. De Souza, Lynn Doran, Atinder Singh, Jeffrey Hansen, Laurie Beth Leonelli, Steven J. Burgess

**Affiliations:** ^1^ Carl R Woese Institute for Genomic Biology University of Illinois Urbana‐Champaign Urbana Illinois USA; ^2^ Department of Agricultural & Biological Engineering University of Illinois Urbana‐Champaign Urbana Illinois USA; ^3^ Department of Plant Biology University of Illinois Urbana‐Champaign Urbana Illinois USA

## Abstract

Improving photosynthesis is proposed as a means of enhancing crop productivity. To assess whether simultaneous increases in light use‐efficiency and carbon assimilation are additive, we investigated the impact of elevated CO_2_ on the performance of a transgenic soybean line engineered for faster relaxation of photoprotection by overexpression of three genes: violaxanthin de‐epoxidase, photosystem II subunit S and zeaxanthin epoxidase (VPZ‐34A). Plants were grown in field plots with and without elevated CO_2._ Physiological and transcriptomic measurements were collected during vegetative and reproductive stages and compared to final biomass and seed production. VPZ‐34A showed improved light use efficiency during vegetative growth, but no increase in carbon assimilation, and no increase in seed or biomass‐related traits under ambient or elevated CO_2_ concentrations. A small number of genes were differentially expressed in VPZ‐34A, including downregulation of a mitochondrial thioredoxin (*Trx o1*) and up‐regulation of two *AP2* transcription factors, which may suggest alterations in redox balance and seed development. In conclusion, the improvements in light use efficiency by modulating photoprotection did not have an additive effect with elevated CO_2_ concentrations and did not stimulate soybean yield in the year measured. Our data highlight the complex relationship between photoprotection, carbon assimilation and yield, and provide avenues for future research.

## Summary statement

Improved light use efficiency of genetically engineered soybean was unaffected under CO2 fertilisation, but it did not translate to improvement in carbon assimilation. CO_2_ fertilisation impacts the expression of photoprotection‐related genes and alters the photoprotection relaxation rate of untransformed soybean.

## 1 Introduction

Approximately 733 million people faced hunger in 2023, and this number has risen since 2019 (FAO IUW and W 2024). Changes in climate, natural disasters and armed conflicts further threaten global food security (Oladipo and Oyinloye 2022; FAO IUW and W 2024; Iriti and Vitalini 2025). Soybeans are the most widely cultivated legume with more than 371 million tons produced worldwide in 2023, making it a key crop from a food security perspective (FAO 2025). The threat of the aforementioned challenges has driven the development of numerous biotechnological approaches to improve crop yield, including soybean (J. A. Anderson et al. 2016; Munaweera et al. 2022; Tyczewska et al. 2023). One of the strategies that has promising results is the improvement of photosynthesis (Simkin et al. 2015; Kromdijk et al. 2016; South et al. 2019; López‐Calcagno et al. 2020; De Souza et al. 2022). However, converting improvements at the leaf level of photosynthesis to crop yield is not straightforward. For example, elevated [CO_2_] (e[CO_2_]) is known to enhance light‐saturated photosynthesis in C_3_ plants by increasing Rubisco carboxylation efficiency and reducing photorespiration through competitive inhibition of oxygenation (Drake et al. 1997; Ainsworth and Long 2005; Bernacchi et al. 2005). However, in soybean, the intensity of the photosynthetic response to e[CO_2_] was shown to be strongly associated with the yield potential of the cultivar evaluated (Ainsworth and Long 2021), highlighting the importance of sink strength. Further, a cross‐scale mathematical model shows that the yield benefit from increasing photosynthetic rates can vary depending on the environmental conditions (Wu et al. 2023). These findings indicate that a greater understanding of how manipulations of photosynthesis interact with whole plant physiology is necessary for the success of engineering approaches.

Several studies have looked to improve the efficiency of light use under fluctuating light (Kromdijk et al. 2016; Garcia‐Molina and Leister 2020; Z. Zhu 2021; De Souza et al. 2022; Lehretz et al. 2022; Milburn et al. 2025). When the supply of photons exceeds demand, such as during high light conditions or drought, build‐up of reduced electron transfer intermediates in the photosynthetic electron transport chain can initiate the production of reactive oxygen species, leading to photodamage and a reduction in photosynthetic activity (J. M. Anderson et al. 1998; Melis 1999; Rutherford et al. 2012). A variety of mechanisms, collectively referred to as non‐photochemical quenching (NPQ) of chlorophyll fluorescence excited states, protect leaves against photodamage by dissipating light energy as heat (Niyogi and Truong 2013; Ruban 2016; Zuo 2025). When light is in excess, protective NPQ is beneficial, and it has been shown to be essential for plant fitness under fluctuating light conditions (Külheim et al. 2002) and during high light stress (X.‐P. Li et al. 2002). However, NPQ can exceed photoprotective requirements depending upon leaf position in the canopy and environmental conditions (Ruban 2017). For example, when leaves transition from full sun to shade, energy dissipation by NPQ can be slow to adjust, and under such transitory conditions, NPQ can divert energy away from photosynthesis (X. G. Zhu et al. 2004; Murchie and Niyogi 2011). This is important, as slow relaxation of NPQ in soybean is predicted to cause an 11% reduction in maximum theoretical daily carbon assimilation (Wang et al. 2020).

Light induces proton pumping across the thylakoid membrane, leading to lumen acidification and build‐up of a proton motive force (*pmf*), which is used to drive ATP synthesis. However, NPQ is also triggered by lumen acidification (Wraight and Crofts 1970; Briantais et al. 1979), and acts as a feedback mechanism, modulating light‐harvesting capacity according to energetic demand. The precise mechanisms of NPQ are debated (for review see Ruban 2016), but, fast energy‐dependent quenching ‘qE’ has been shown to be related to acidification of the lumen, triggering a conformational change of photosystem II (PSII)‐associated antennae via protonation of PSII subunit S (PsbS) in plants whereas green algae employs protonation of light harvesting complex stress‐related proteins to quench excess excitation of chlorophyll (X.‐P. Li et al. 2000; Peers et al. 2009; Johnson et al. 2011; Liguori et al. 2013; Wilson et al. 2024). In addition, lumen acidification activates violaxanthin de‐epoxidase (VDE), which catalyses the conversion of violaxanthin (Vx) to zeaxanthin (Zx) via antheraxanthin (Yamamoto et al. 1962; Hager 1969; Yamamoto and Kamite 1972), with accumulation of Zx involved in modulation of quenching (Demmig‐Adams et al. 1996; Niyogi et al. 1998). An intermediate relaxing component of NPQ, termed ‘qZ’ is associated with the catabolism of zeaxanthin, and typically operates on the time scale of 10–15 min (Niyogi et al. 1998; Nilkens et al. 2010). This conversion of Zx back to Vx, is catalysed by the stromal localised enzyme zeaxanthin epoxidase (ZEP) (Sapozhnikov 1957; Siefermann and Yamamoto 1975; Marin et al. 1996) and is involved in relaxing NPQ.

Following model predictions, Kromdijk et al. (2016) overexpressed homologues of *VDE*, *PsbS* and *ZEP* genes from Arabidopsis (*AtVPZ*) in tobacco, to speed up relaxation of NPQ and reduce the loss of potential energy during shadeflecks. This was found to lead to an increase in light use efficiency under fluctuating light, which translated to greater biomass production in small‐scale field experiments (Kromdijk et al. 2016). A follow‐up study on soybean applied the same strategy and observed accelerated response of the fast (τ_1_; qE) and intermediate (τ_2_; qZ) time constants for NPQ relaxation upon transition from sun to shade by 37% and 67%, respectively (De Souza et al. 2022). This resulted in an improvement in photosynthetic efficiency under fluctuating light and an average increase of 24.5% in seed production compared to untransformed soybean in one out of 2 years of experiments (De Souza et al. 2022). Similar efforts in other species have resulted in reduced biomass and/or yield, including Arabidopsis (Garcia‐Molina and Leister 2020), potato (Lehretz et al. 2022), and creeping bentgrass (Z. Zhu 2021), whereas in Setaria, improvement in yield under specific conditions was observed (Milburn et al. 2025). Therefore, the particular conditions under which this modification is likely to be beneficial are not clearly understood, necessitating further investigation.

A variety of modelling approaches have assessed the potential impact of combining improvements in CO_2_ fixation and light reactions. Typically, these models predict an additive effect, although the precise scale of improvement and influence of environment vary (Wu et al. 2023). Improved response from additive effects also finds some support from experiments in tobacco involving overexpression of SBPase and an algal cytochrome b_6_f protein (López‐Calcagno et al. 2020). Given the promising findings of De Souza et al. (2022) and conflicting reports in the literature, we sought to test whether stacking increases in light use efficiency with increased CO_2_ fixation would lead to additive gains and which transcription scale changes underpin the observed physiology. Therefore, we characterised the physiological and transcriptional responses of a wild‐type and a transgenic VPZ soybean line under field conditions, comparing growth under elevated [CO_2_] levels as a proxy for increased carbon assimilation.

## 2 Materials and Methods

### 2.1 Field Site, Plant Material and Experimental Design

This study was conducted during the summer of 2021 at the Soybean Free‐Air CO_2_ Enrichment (SoyFACE) facility in Champaign, IL, USA (https://soyface.illinois.edu/) as described in Aspray et al. (2023). The soil types of the experimental plots were Drummer silty clay loam and Flanagan silt loam (Figure S1) (USDA 2026). Air temperature, precipitation, solar radiation and ambient [CO_2_] data were collected as daily averages as described in Aspray et al. (2023) (Figure S2A–D). Vapour pressure deficit was calculated using the August‐Roche‐Magnus Equation (Figure S2E) (Alduchov and Eskridge 1996).

*Glycine max* cv. Maverick; ND‐18‐34A, (hereafter named VPZ‐34A), overexpressing three genes from *A. thaliana*: VDE (*AtVDE)*, PSII subunit S (*AtPsbS*) and ZEP (*AtZEP*) was used in this study. This transgenic line was selected due to its higher seed production in previous experiments (De Souza et al. 2022). T5 seeds from the transgenic line were obtained as previously described (De Souza et al. 2022) and planted paired with a control of non‐transgenic seeds (WT) from the same cultivar.

The experiment had four experimental blocks. Each block consisted of one plot that was fumigated to elevate the [CO_2_] to 600 µmol mol^−1^ paired with another plot that served as a control with the current a[CO_2_] concentration of 410 µmol mol^−1^. Each plot (7 m^2^) was split into two sub‐plots to accommodate four rows of 1.2 m for each line. Each row had 34 seeds, totalling 136 plants per sub‐plot. Row spacing was 0.75 m, and seed spacing 3.8 cm. Seed planting depth was 3.5 cm. The experiment had a 10 m bare border to comply with APHIS/USDA regulatory permits. All the measurements and tissue sampling were done in the middle two rows of each sub‐plot.

A few days before the planting, the field was cultivated and tilled. On 8 June 2021, T5 seeds from VPZ‐34A and WT were hand‐planted. Prior to planting, seeds were treated with CruiserMaxx Vibrance to protect against damage from early‐season insects and diseases. Diatomaceous earth (PF Harris, Cartersville, GA, USA) was added 8 days after sowing (DAS) to prevent cutworms. For pest control, the broad‐spectrum insecticide Mustang Maxx (FMC Corporation, Philadelphia, PA, USA) was applied during the V4–V5 stage following the manufacturer's instructions. To allow homogeneous germination, irrigation was supplied manually until the emergence of seedlings. After that stage, the experiment was rainfed.

### 2.2 Measurement of NPQ

The speed of NPQ relaxation during the transition from high light to low light was assessed using a chlorophyll fluorescence imager (CF Imager, Technologica Ltd., UK) following the protocol described by Gotarkar et al. (2022). Leaf disks were sampled at 32 DAS during the V3–V4 stage. Time constants related to qE (τ_1_), qZ (τ_2_) and NPQ at steady‐state high light (NPQ_max_) were calculated by fitting a sum of double exponential curves to the time‐course of NPQ relaxation after transition from high (15 min of 2000 photosynthetic photon flux density [PPFD]) to low light (50 min of 50 PPFD) (Dall'Osto et al. 2014; Long et al. 2022).

### 2.3 Gas Exchange Measurements

During vegetative (V4–V5, 29–30 DAS) and reproductive (R5–R6, 93–94 DAS) stages, CO_2_ assimilation response curves to light were measured simultaneously with chlorophyll fluorescence following the protocol described by De Souza et al. (2022) for NPQ activation and fluctuating light conditions. Theoretical maximum quantum efficiency of CO_2_ assimilation (ΦCO_2, max_) and theoretical maximum quantum efficiency of linear electron transport (ΦPSII_max_) were calculated as the slope of carbon assimilation (*A*) over quanta and electron transport rate (ETR) over quanta from 50 to 200 µmol m^−2^ s^−1^. Non‐photochemical quenching during low light (NPQ_low‐light_), photochemical quenching (qP, *F*_q_ *'/F*_v_ *'*), maximum efficiency of PSII photochemistry in the light if all reaction centers were open (*F_v_* '/*F_m_* '), and instantaneous quantum yield of linear electron transport (ΦPSII, *F*_q_ *'*/*F*_m_ *'*), were averaged over the same time points. Averages for NPQ during high light, ETR and A were also calculated for the light intervals greater than 1800 PPFD during the fluctuating light protocol. Measurements were done on the youngest fully expanded leaves using a portable gas exchange system with a cuvette integrated with a modulated chlorophyll fluorometer and light source (LI‐6800; LI‐COR, Lincoln, NE, USA).

The maximum apparent carboxylation rate by Rubisco (*V*_c, max_) and the maximum regeneration of ribulose‐1,5‐bisphosphate expressed as ETR (*J*_max_) under steady‐state conditions were calculated from fitting *A*/intercellular [CO_2_] (*C*_i_) curves obtained using the LI‐6800 as described above. Measurements were taken at vegetative (V4–V6, 37 DAS) and reproductive (R4–R5, 86 DAS) stages. For *A*/*C*_i_ curves, the light intensity was kept constant to 2000 µmol m^−2^ s^−1^ (90% red, 10% blue), block temperature at 28°C, relative humidity inside the chamber at 60% ± 2%, and the reference CO_2_ concentration was varied following the sequence: 400, 300, 200, 150, 100, 75, 50, 400, 400, 600, 800, 1000, 1200, 1500, 1800 and 2000 µmol mol^−1^ for plants grown at a[CO_2_] after Long (2003) and 600, 400, 300, 200, 150, 100, 75, 50, 600, 600, 800, 1000, 1200, 1500, 1800, 2000 µmol mol^−1^ for plants grown at e[CO_2_] per (Ainsworth et al. 2007). Prior to fitting the curves, values were adjusted for temperature response to 25°C according to Bernacchi et al. (2001) and McMurtrie and Wang (1993).

Diurnal measurements of photosynthesis were collected using the previously described LI‐6800 at 44 DAS during the vegetative stage (V6–V7) at 5 AM, 8 AM, 11 AM, 2 PM, 5 PM and 8 PM. The reference CO_2_ of 400 ppm was used for measurements from a[CO_2_] and 600 ppm for e[CO_2_]. The leaf chamber fan speed was set to 10 000 rpm, flow rate to 400 µmol s^−1^, relative humidity to 60%, and the light source was configured to 90% red and 10% blue. Incident light provided to the leaf and leaf temperature controls were adjusted throughout the day to match environmental conditions. Leaves were allowed to reach steady state *A* and stomatal conductance (*g*_s_) in the chamber before ΦPSII, NPQ, *C*_i_, *A* and *g*_s_ measurements were logged. Intrinsic water use efficiency was calculated as *A* divided by *g*_s_ (Osmond et al. 1980). Leaves from three independent plants were sampled and averaged to represent a plot.

### 2.4 Pigment Extraction and HPLC Analysis

Leaf tissue for pigment analysis was collected on the same day during the same time points as the diurnal photosynthesis measurements. Using a cork borer, three 13.4 mm leaf disks from the youngest fully expanded leaf of three different plants within a plot were sampled and pooled together. Samples were placed in 2 mL nuclease‐free tubes, immediately frozen in liquid nitrogen and stored at −80°C. Samples were ground to a fine powder with 4 mm SPEX stainless steel grinding beads (2150; SPEX SamplePrep, USA) using TissueLyser Universal Laboratory Mixer‐Mill disruptor (85210; QIAGEN, Germany), at 24 Hz, twice for 1.5 min, stopping to cool cassettes in liquid N_2_. Pigments were extracted from ground tissue using 2 × 100% acetone as previously described in Leonelli (2022). Pigment composition and quantification were performed using the method described in Müller‐Moulé et al. (2002) with an Agilent 1290 Infinity UPLC system. Pigment concentrations were normalised to chlorophyll *a* to facilitate comparisons across plots. De‐epoxidation state (DES) was calculated as follows: for α‐xanthophylls, Lut/(Lut + Lx); for β‐xanthophylls, (Zea + 0.5Ant)/(Vio + Ant + Zea).

### 2.5 Measurement of Growth and Development

Plant height and number of nodes were measured from 24 DAS until 85 DAS, when the plants reached the R5–R6 stage. Plant height was measured from the cotyledon scar to the growing point. The stages of soybean development were evaluated weekly starting at 8 DAS until the end of the experiment using the Fehr and Caviness (1977) method. Leaf area index (LAI) was measured on 45, 50, 57, 63, 72, 78, 86, 93 and 107 DAS using a portable plant canopy analyzer (LAI‐2200C; Li‐Cor, Lincoln, NE, USA) at ground level between the middle two rows of each plot. Stem biomass and seed weight were determined after harvesting once the plants reached the R8 stage of development. Harvest was done on 7 October 2021, at 121 DAS. Ten plants of each plot were collected and placed in individual paper bags. Total number of pods, number of seeds per pod and stem biomass were determined after drying the plants in an oven at 60°C until constant weight. Harvest index was calculated as the ratio of seeds to the total above‐ground biomass. Seed biomass per plot was determined by hand harvesting and threshing (LD350 Laboratory Thresher, Wintersteiger, Austria) all remaining plants per plot.

### 2.6 RNA‐Seq Analysis

Leaf samples were collected at 31 DAS and 86 DAS, during V4–V5 and R5–R6 stages, respectively, between 9:00 and 11:00 AM. Samples were collected, stored and ground as described under pigment extraction.

Total RNA was extracted using the RNeasy Plant Mini Kit (74904; Qiagen, Germany) according to the manufacturer's protocol with the following modification: RPE buffer wash was increased from one to 4–5 times. Further, the total RNA sample was digested with RNase‐Free DNase Set (79254; Qiagen, Germany) to remove any traces of genomic DNA contamination. Total RNA concentration was initially evaluated using a NanoDrop One Microvolume UV‐Vis Spectrophotometer (Thermo Scientific, USA). The quality of each RNA sample was determined in 1.2% agarose‐TAE (0.5×) gel electrophoresis containing SYBR Safe DNA Gel Stain (Thermo Fisher, USA) by evaluating the integrity of the 28S and 18S ribosomal RNA bands and the absence of smears. Construction of libraries and sequencing using the Illumina NovaSeq 6000 system (Illumina, CA, USA) were performed at the Roy J. Carver Biotechnology Center at the University of Illinois at Urbana‐Champaign. Purified total RNAs were run on a Fragment Analyzer (Agilent, CA, USA) to re‐evaluate RNA integrity. The total RNAs were converted into individually barcoded polyadenylated mRNAseq (PolyA) libraries with the Kapa Hyper stranded mRNA Sample Prep kit (08098123702; Roche, IN, USA). Libraries were barcoded with Unique Dual Indexes, which have been developed to prevent index switching. The adaptor‐ligated double‐stranded cDNAs were amplified by PCR for eight cycles with the Kapa HiFi polymerase (07958927001; Roche, IN, USA). The final libraries were quantified with Qubit (ThermoFisher, MA, USA), and the average cDNA fragment sizes were determined on a Fragment Analyzer. The libraries were diluted to 10 nM and further quantified by qPCR on a CFX Connect Real‐Time qPCR system (Biorad, CA, USA) for accurate pooling of barcoded libraries and maximisation of the number of clusters in the flowcell.

The barcoded RNA‐SEQ libraries were loaded on two SP lanes for 101 cycles on a NovaSeq 6000 sequencing system. The libraries were sequenced from one end of the fragments for a total of 100 bp. The Fastq read files were generated and demultiplexed with the bcl2fastq v2.20 Conversion Software (Illumina, CA, USA). Raw reads were deposited in the NCBI Sequence Read Archive, BioProject accession GSE270020. Reads from 32 RNA‐SEQ libraries were first filtered to remove low‐length reads (i.e., reads < 34). The reads were aligned to the soybean genome Wm82.a4.v1 (downloaded from Phytozome [Sreedasyam et al. 2023], Phytozome genome ID: 508, version: *Gmax_Wm82_a4_v1* [Valliyodan et al. 2019]) using the STAR aligner (Dobin et al. 2013) at default parameters for Arabidopsis. The sequence of the three Arabidopsis VPZ genes (VDE (*AtVDE, AT1G08550*), PSII subunit S (*AtPsbS, AT1G44575*) and ZEP (*AtZEP, AT5G67030*) (genome version TAIR10, annotation version Araport11) was added to the soybean genome before aligning reads to the soybean genome. Furthermore, the *featureCounts* programme from the subreads package was used to count the number of reads assigned to each gene (Liao et al. 2014). Reads with multi‐align flag were assigned fractionally to each of the mapped loci.

Four replicates for each treatment‐genotype combination were sampled from the four blocks/rings in the field. To remove batch effect associated with the blocks/rings, the ComBat‐seq programme was used (Zhang et al. 2020). Differential gene expression (DGE) from the V4–V5 stage and the R5–R6 stage was analysed separately. DGE analysis was conducted with the limma package (Ritchie et al. 2015). The low count genes were filtered out, and libraries were normalised for size using the Trimmed Mean of *M*‐values normalisation method (Robinson and Oshlack 2010). Differential expression analysis was carried out by fitting a linear model. Design matrix was set up to evaluate the statistical significance of main effects (CO_2_ and line) and interaction effect (CO_2_ × line). False discovery rate was controlled using the Benjamini and Hochberg method. Adjusted *p*‐values were obtained from ‘p. adjust‘ function from stats package in R. For main effects, genes with *p*‐adjusted < 0.05 and log_2_ fold change (FC) > 1 were considered to be statistically significant unless stated otherwise. For the interaction effect, genes with p‐adjusted < 0.05 were considered significant. Genes significant for the interaction term were clustered into co‐expressed clusters to identify dominant interaction types. Clustering was performed using the hierarchical clustering method. CO_2_ and line effect on predefined gene sets (KEGG pathways) was assessed using gene set enrichment analysis (GSEA). For both main effects, the list of expressed genes was sorted based on the log_2_FC separately. WT and a[CO_2_] were considered the control condition for the analysis. ‘gseKEGG’ function of ClusterProfiler R package was used to run the analysis (Yu et al. 2012). Top 15 pathways with *p*‐adjusted values < 0.05 were considered significant.

A separate statistical analysis was conducted to test the effect of the growth stage, CO_2_ and line on the native and transgenic *ZEP*, *VDE* and *PsbS* genes. For this analysis, samples for two growth stages were analysed together. DGE analysis was performed using the steps described above. Model design consisted of three main effects (the growth stage, CO_2_ and line) and their interactions. Raw *p*‐values obtained for each main effect and interaction effect were corrected for multiple tests using the Benjamini‐Hochberg procedure as described above. Log_2_FC was calculated for each treatment, growth stage and line group. Genes with *p*‐adjusted value < 0.05 and Log_2_FC > 1 were considered statistically and biologically significant.

### 2.7 Statistical Analysis

Data from physiological measurements were analysed using the JMP Software (JMP Pro 15.0.0, SAS Institute Inc.) or R software (R Core Team 2025). Normal data distribution was checked using the Shapiro–Wilk *W* test, and equal variances using Brown–Forsythe and Levene tests (Dag et al. 2018; Levene 1960; Shapiro and Wilk 1965; Brown and Forsythe 1974; Royston 1982). Where required to meet the assumption of normality, a Box–Cox transformation was applied. Significant differences were determined using a mixed model with restricted maximum likelihood considering [CO_2_] treatment, line and the interaction between [CO_2_] and line as fixed effects, and block as a random effect (Cheung 2013). For diurnal gas exchange measurements or measurements taken in different stages of development, the times or dates were considered repeated measurements. Diurnal pigment data were analysed using generalised linear mixed models assuming a gamma distribution (Brooks et al. 2017). The DHARMa package in R was used to assess the dispersion, outliers and uniformity of residuals (Hartig 2026). Four extreme outliers from violaxanthin and V + A + Z data were removed based on the sample distribution. Where the assumption of normality was not met, the non‐parametric Kruskal–Wallis test was used (Hollander et al. 2015). Significant differences were considered when *p* ≤ 0.1. That significant threshold was used to minimise the possibility of a type II error given the low replication level (*n* = 4).

## 3 Results

### 3.1 Faster NPQ Relaxation and Higher Photosynthetic Efficiency of VPZ‐34A Soybean Is Not Affected by Elevated [CO_2_] but Is Affected by Growth Stage

**Figure 1:**
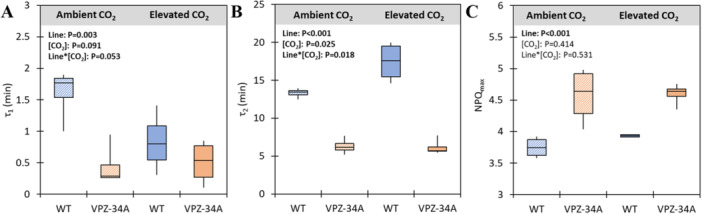
Relaxation time constants for (A) fast (τ_1_) and (B) medium (τ_2_) NPQ components, and (C) maximum NPQ (NPQ_max_) of WT and VPZ‐34A grown at SoyFACE, Champaign, IL, USA under ambient and e[CO_2_]. Significant differences between lines, [CO_2_] and interaction line*[CO_2_] are in bold and indicated by *p* < 0.1 (*n* = 4).

The time constants related to the fast (τ_1_; qE) and intermediate (τ_2_; qZ) relaxing components of NPQ in VPZ‐34A were significantly lower than those of the WT in both [CO_2_] conditions (Figure 1A,B). While e[CO_2_] did not affect τ_1_ and τ_2_ in VPZ‐34A, it reduced τ_1_ in WT by 49% and increased τ_2_ by 31% (Figure 1A). Maximum NPQ was higher in the VPZ‐34A and remained unaffected by [CO_2_] (Figure 1C).

**Figure 2:**
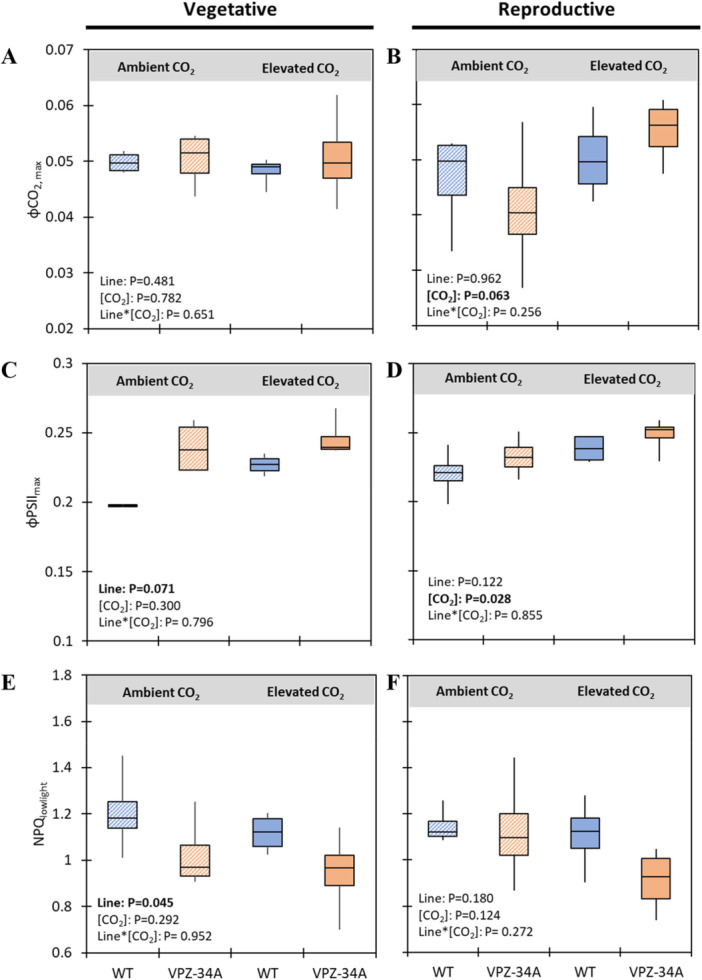
Maximum quantum efficiency of CO_2_ assimilation (ΦCO_2, max_) (A, B), maximum quantum efficiency of linear electron transport (ΦPSII_max_) (C, D) and non‐photochemical quenching during sun to shade transitions (NPQ_lowlight_) (E, F) under fluctuating light of WT and VPZ‐34A grown at SoyFACE, Champaign, IL, USA, under ambient and elevated a[CO_2_]. Values represent repeated measurements from vegetative (V4–V5) (A, C, E) and reproductive (R5–R6) (B, D, F) stages. Significant differences between lines, [CO_2_] and interaction line*[CO_2_] are in bold and indicated by *p* < 0.1 (*n* = 4). [Color figure can be viewed at wileyonlinelibrary.com]

Under fluctuating light, the theoretical maximum quantum efficiency of linear electron transport (ΦPSII_max_) and non‐photochemical quenching during low light (NPQ_low‐light_, 50–200 µmol m^−2^ s^−1^) were statistically different between VPZ‐34A and WT only in the vegetative stage. ΦPSII_max_ in VPZ‐34A was, on average, 7% and 5% greater than in WT at stages V4‐5 and R5–R6, respectively (Figure 2C,D). NPQ_low‐light_ in VPZ‐34A was 15% lower than WT at V4–V5 and it dropped to 10% lower than WT at R5–R6 under ambient conditions, a slight decrease was observed under e[CO_2_] but this was not significant (Figure 2E,F). No significant differences in theoretical maximum quantum efficiency of carbon assimilation (ΦCO_2, max_) were observed between genotypes (Figure 2A,B). Both genotypes exhibited reduced NPQ_low‐light_ and increased ΦPSII_max_ under e[CO_2_] compared to those grown at a[CO_2_]. At e[CO_2_], ΦPSII_max_ for both WT and VPZ‐34A was higher than at a[CO_2_] with a 4% gain at V4–V5 and a 7% gain at R5–R6 (Figure 2C,D). NPQ_low‐light_ also showed greater reduction under e[CO_2_] with an 8% decrease in V4–V5 and 11% reduction at R5–R6 as compared to a[CO_2_] (Figure 2E,F). However, there was no significant interaction between VPZ‐34A and e[CO_2_] for any of those measurements. Under NPQ activation, no significant changes in ΦPSII_max_ or ΦCO_2, max_ between VPZ‐34A and WT were observed (Figure S3; Figure S4A–D). However, a significant difference in ΦPSII_max_ between ambient and elevated [CO_2_] was observed with a 3% reduction in e[CO_2_] at V4–V5 and a 3% increase in e[CO_2_] at R5–R6 (Figure S4C,D). ΦCO_2, max_ also showed a treatment effect with a 7% loss at e[CO_2_] at V4–V5 and a line by treatment effect at R5–R6 with a 10% increase in ΦCO_2, max_ for VPZ‐34A at e[CO_2_] and a 10% decrease for WT at e[CO_2_] (Figure S4A,B). NPQ_low‐light_ values were 13% lower in VPZ‐34A in both [CO_2_] across both growth stages (Figure S4E,F). There was no significant line effect of line on mean assimilation during high light in fluctuating light at either growth stage (Figure S8E,F). There was no significant change in *V*_c, max_ and *J*_max_ between WT and VPZ‐34A, but the seasonal average of *V*_c, max_ was 23% lower in WT and 14% lower in VPZ‐34A at e[CO_2_] as compared to a[CO_2_] (Table 1).

**Table 1:** Maximum rate of carboxylation by Rubisco (*V*_c, max_; µmol m^−^^2^ s^−1^) and maximum rate of photosynthetic electron transport (*J*_max_; µmol m^−2^ s^−1^) at steady‐state in WT and VPZ‐34A grown at SoyFACE, Champaign, IL, USA under ambient and e[CO_2_]. Values represent repeated measurements from vegetative (V4–V5) and reproductive (R5–R6) stages. Values are mean ± SE (*n* = 4).

Season averages							
	Ambient CO_2_		Elevated CO_2_		ANOVA results		
	WT	VPZ‐34A	WT	VPZ‐34A	Line	[CO_2_]	Line* [CO_2_]
*V*_cmax_	70.47 ± 6.57	66.08 ± 5.91	54.24 ± 5.96	57.79 ± 4.95	0.970	**0.031**	0.465
*J*_max_	125.99 ± 7.52	122.59 ± 9.67	115.21 ± 11.19	126.24 ± 7.83	0.691	0.703	0.443
Vegetative (V4–V5)							
*V*_cmax_	83.08 ± 5.37	70.95 ± 5.23	59.18 ± 7.17	62.32 ± 6.10	0.501	**0.033**	0.269
*J*_max_	126.48 ± 5.38	117.61 ± 7.38	100.68 ± 9.71	122.34 ± 9.69	0.458	0.233	**0.097**
Reproductive (R5–R6)							
*V*_cmax_	57.85 ± 8.15	61.21 ± 10.95	49.30 ± 9.89	53.25 ± 7.98	0.687	0.378	0.974
*J*_max_	125.50 ± 15.31	127.58 ± 19.12	129.74 ± 18.7	130.13 ± 13.5	0.945	0.851	0.967

*Note:* Significant differences between lines, [CO_2_] and interaction line*[CO_2_] are in bold and indicated by *p* < 0.1.

In summary, expression of *AtVPZ* increased maximum NPQ and sped up the rate of relaxation in VPZ‐34A relative to WT. As expected, there was no change in steady state assimilation or electron transport; however, while *AtVPZ* expression resulted in increased light use efficiency under fluctuating irradiance, this did not translate to an increase in the efficiency of carbon assimilation. Interestingly, the relative benefit of *AtVPZ* on photosynthesis was limited to vegetative stages, due to developmental effects on NPQ relaxation kinetics, and growth under e[CO_2_] altered the kinetics of NPQ relaxation in WT plants, but had no impact on VPZ‐34A.

### 3.2 e[CO_2_] Increases Zeaxanthin Accumulation Without Altering Steady State NPQ and Photosynthesis in Both WT and VPZ‐34A Lines

**Figure 3:**
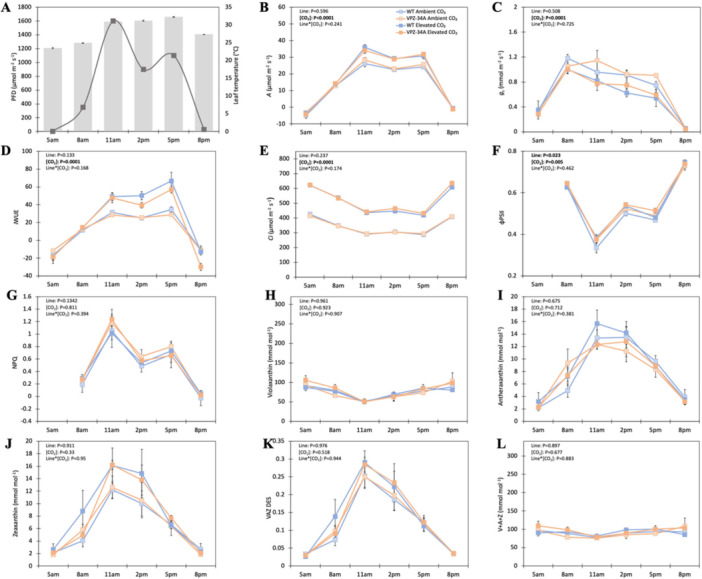
Diurnal measurements of photosynthesis were conducted under light levels and leaf temperatures commensurate with environmental conditions (A). CO_2_ assimilation (A) (B), stomatal conductance (*g*_*s*_) (C), intrinsic water use efficiency (*i*WUE) (D), intracellular [CO_2_] (*C*_*i*_) (E), instantaneous ΦPSII (F), NPQ (G) and violaxanthin (V) (H), antheraxanthin (A) (I), and zeaxanthin (Z) (J) levels as normalised by chlorophyll a content (mmol mol^−1^) were measured across six time points in WT and VPZ‐34A grown at SoyFACE, Champaign, IL, USA under ambient and elevated a[CO_2_]. The de‐epoxidation state (DES) related to the VAZ cycle is represented in fraction and calculated as (Z + 0.5 A)/(V + A + Z) (K), and the total pigment content in the VAZ cycle is calculated as (V + A + Z) (L). Significant differences between lines, [CO_2_] and interaction line*[CO_2_] are in bold and indicated by *p* < 0.1 (*n* = 4). [Color figure can be viewed at wileyonlinelibrary.com]

To investigate the response of WT and VPZ‐34A to e[CO_2_] in situ, we conducted a diurnal assessment of photosynthesis and xanthophyll cycle pigments on vegetative stage plants (V6–V7) (Figure 3). Light intensity increased from dawn (5 PM) to 11 AM, followed by a decline at around 2 PM due to cloud cover, before rising again in midafternoon and decreasing towards dusk (Figure 3A). Leaf temperatures rose throughout the day from ~25°C at dawn peaking around 3°C at 5 PM before decreasing towards 8 PM (Figure 3A). As expected, when grown at e[CO_2_], WT and VPZ‐34A plants showed higher carbon assimilation and reduced stomatal conductance, resulting in higher intrinsic water use efficiency (iWUE) due to increased carboxylation and stomatal closure in response to increased intercellular [CO_2_] *C*_i_ (Figure 3B–E). This was accompanied by a slight increase in ΦPSII in both genotypes grown under e[CO_2_] relative to ambient conditions, with values tracking changes in light intensity throughout the day (Figure 3F). Intriguingly, there was also a slight increase in ΦPSII in VPZ‐34A relative to WT irrespective of conditions (Figure 3F). This was accompanied by steady state NPQ values trending higher in VPZ‐34A, although the differences were not deemed significant between lines or CO_2_ concentrations (Figure 3G).

Pigment profiles were assessed to determine whether changes in photosynthesis were reflected in changes in pigment concentrations. Violaxanthin levels remained largely unchanged between lines and growth conditions (Figure 3H). Antheraxanthin levels were slightly higher in VPZ‐34A at 8 AM (Figure 3I), without a corresponding increase in Zeaxanthin (Figure 3H), but otherwise remained largely consistent between lines and conditions (Figure 3I). Zeaxanthin content did trend higher for plants grown at e[CO_2_] during the middle of the day, which also reflected in an elevated DES of xanthophylls (VAZ DES) (Figure 3J,K). However, these changes were not significant, and there were no overall changes to total pigment contents (Figure 3L).

Taken together, these data indicate that growth at CO_2_ increased carbon assimilation, water use and ΦPSII irrespective of genotype. While there were potentially minor differences in pigment contents and steady state NPQ in VPZ‐34A plants, these differences did not reach statistical significance, which would be consistent with the trait predominantly influencing dynamic behaviour.

### 3.3 The Response of Plant Growth and Development to Elevated [CO_2_] Is Similar Between WT and VPZ‐34A

**Figure 4:**
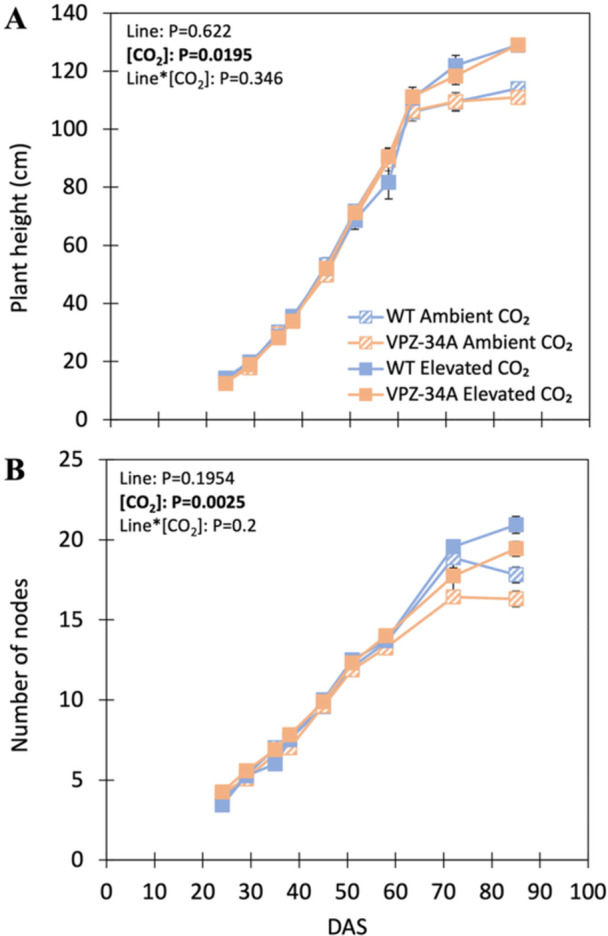
Plant height (cm) (A) and number of nodes (B) of WT and VPZ‐34A grown at SoyFACE, Champaign, IL, USA under ambient and e[CO_2_]. Values are mean ± SE (*n* = 4). DAS = days after sowing. Significant differences between lines, [CO_2_] and interaction line*[CO_2_] are in bold and indicated by *p* < 0.1. [Color figure can be viewed at wileyonlinelibrary.com]

At 85 DAS, there were no significant differences in height between growth conditions or genotypes (Figure 4A). At e[CO_2_], WT plants had on average three more nodes than at a[CO_2_], while the VPZ‐34A had on average only two more nodes (Figure 4B). While the growth stages did not differ between VPZ‐34A and WT under ambient or e[CO_2_] during the vegetative stages (VE to V8), most VPZ‐34A plants reached the R8 stage at e[CO_2_] faster than WT (DAS 126; Figure S10).

**Figure 5:**
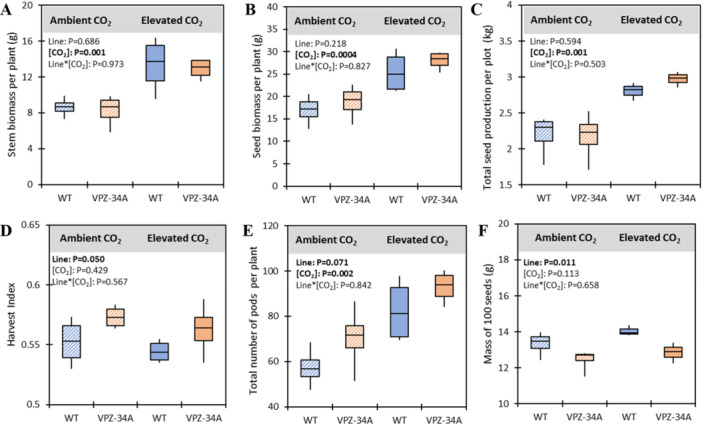
Stem (A) and seed (B) biomass per plant, seed biomass per plot (C), harvest index (D), total number of pods per plant (E) and mass of 100 seeds (F) of WT and VPZ‐34A grown at SoyFACE, Champaign, IL, USA under ambient and e[CO_2_]. Significant differences between lines, [CO_2_] and interaction line*[CO_2_] are in bold and indicated by *p* < 0.1 (*n* = 4). [Color figure can be viewed at wileyonlinelibrary.com]

At harvest, stem and seed biomass per plant were 50%–55% greater at e[CO_2_] compared to a[CO_2_], with no significant differences between lines (Figure 5A,B). Similarly, seed production per plot was not altered between WT and VPZ‐34A, but plots under e[CO_2_] produced significantly more seeds than in ambient a[CO_2_] (Figure 5C). Despite no differences in seed biomass per plant or per plot, the harvest index of VPZ‐34A plants was 3% greater than that of WT (Figure 5D). Harvest index was not affected by [CO_2_] (Figure 5D). Seeds in the VPZ‐34A were overall 8% smaller than the WT (Figure 5F).

### 3.4 VDE, ZEP and PsbS Gene Expression Were Significantly Affected by the Growth Stage

*AtVDE*, *AtZEP* and *AtPsbS* were transcriptionally active in all transgenic samples. Their expression in the reproductive stage was generally lower than in the vegetative stage. This growth stage effect was statistically significant for *AtVDE* and *AtZEP*, but not for *AtPsbS* (File S1). However, growth under e[CO_2_] did not significantly affect the expression of transgenes (Figure S11). Gene expression of seven native VPZ genes (*GmZEP*, *GmVDE* and *GmPsbS*) responded to various independent variables and their interactions (Figure S11). However, only the growth stage resulted in a statistically significant change (i.e., FDR < 0.05 and log_2_FC > 1) for two *GmZEP* genes (*Glyma.09G00600* and *Glyma.11G055700*). Among the two *GmZEP* genes *Glyma.09G00600*'s expression was roughly eight times greater across both lines and [CO_2_] at the R5–R6 stage compared to V4–V5 stage. Upregulation of *Glyma.11G055700* at the R5–R6 stage ranged from one to four‐fold.

### 3.5 CO_2_ Treatment and VPZ Overexpression on Global Transcription During Vegetative and Reproductive Stages

**Figure 6:**
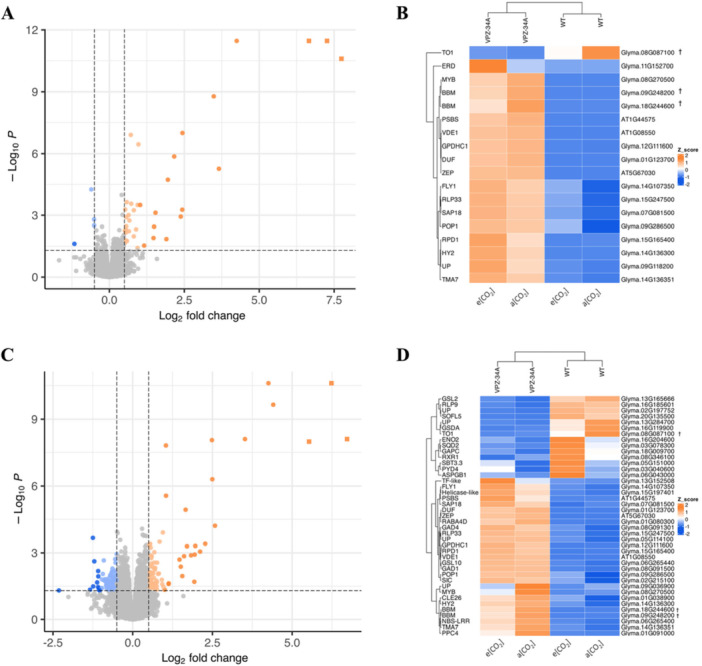
Main genotype effects differentially expressed genes (DEGs) at two growing stages. (A, C) Volcano plots representing DEGs between the vegetative stage and the reproductive stage, respectively. Circles represent soybean genes, squares represent transgenes. Darker shade of colour represents significant genes with log2FC > 1 (B, D). Heatmaps of significant DEGs (log2FC > 1) at the vegetative stage and the reproductive stage, respectively. ‘†’ highlights the gene IDs mentioned in text. [Color figure can be viewed at wileyonlinelibrary.com]

**Figure 7:**
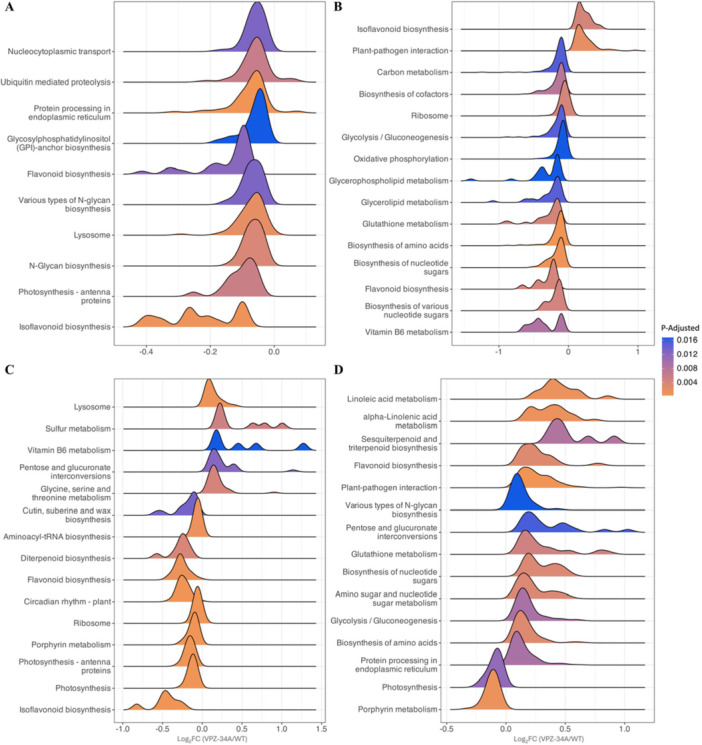
Gene set enrichment analysis (GSEA) of expressed genes at two growing stages using KEGG pathways as gene sets. (A, B) GSEA using log_2_FC calculated with VPZ‐34A as treatment and WT as control at V4–V5 stage (A) and R6 stage (B). (C, D) GSEA using log_2_FC calculated with e[CO_2_] as treatment and a[CO_2_] as control at V4–V5 stage (C) and R6 stage (D). The colour represents the statistical significance of the enriched pathway. The density plots for each pathway represent the distribution of log_2_FC values of genes within that pathway. [Color figure can be viewed at wileyonlinelibrary.com]

At V4–V5 stage, 15 genes were identified as differentially expressed genes (DEGs) between WT and VPZ‐34A, while 35 genes were differentially expressed at the R5–R6 stage (FDR < 0.05 and log_2_FC > 1) (File S1). Notably, 13 of the 15 DEGs from the V4–V5 stage were also differentially expressed at R6, indicating a consistent transcriptional signature associated with the VPZ cassette. Among the genes consistently differentially expressed across both stages, the most notable was the downregulation of *Glyma.08G087100* in VPZ‐34A samples (Figure 6B,D). This gene is a homologue of a mitochondria‐localised thioredoxin *Trx o1* in Arabidopsis. In addition, two ethylene responsive factors—*Glyma.09G248200* and *Glyma.18G244600*—which are homologous to the *BABY BOOM* (BBM) gene in Arabidopsis, were upregulated in VPZ‐34A samples (Figure 6B,D). Given the small number of DEGs observed, GSEA was used to assess the entire list of expressed genes to delineate any weaker but pathway‐wide effects. GSEA revealed that flavonoid and isoflavonoid biosynthetic pathways were enriched at both growing stages for genotype effect (Figure 7A,B). In addition, enriched pathways at V4–V5 included protein processing, N‐glycan biosynthesis and photosynthesis antenna proteins (Figure 7A). Similarly, at R5–R6, pathways such as carbon metabolism, oxidative phosphorylation, proteasome and glutathione metabolism were enriched. However, the range of log_2_FC among these pathways was less than one (Figure 7A,B). Thus, log_2_FC threshold for DEGs was relaxed to 0.5 to identify significant DEGs among the pathways identified by GSEA. At log_2_FC > 0.5, we identified 42 DEGs (38 upregulated and four downregulated in VPZ‐34A) at vegetative stage and 168 DEGs (85 upregulated and 83 downregulated) at R5–R6 stage. The relaxed log_2_FC cutoff allowed identification of three carbon metabolism related DEGs at R5–R6, in addition to *Glyma.03G040600* (Pyrimidine 4, *PYD4*) which was significantly downregulated in VPZ‐34A at log_2_FC > 1 cutoff. *Glyma.01G091000* (phosphoenolpyruvate carboxylase 4, *PPC4*) was upregulated, *Glyma.16G204600* (Enolase 2, *ENO2*) and *Glyma.18G009700* (glyceraldehyde‐3‐phosphate dehydrogenase C subunit 1, *GAPC1*) were downregulated in VPZ‐34A (File S1). Apart from carbon metabolism, glutathione metabolism at the reproductive stage and N‐glycan biosynthesis at the vegetative stage were among the pathways identified via GSEA that consisted of two and one DEGs, respectively, after log_2_FC cutoff relaxation. *Glyma.02G069500*, a flowering locus T 2c (*FT2c*) was another development regulation gene identified as DEG (at log_2_FC > 0.5) in addition to the above‐mentioned *BBM* genes, but only at the V4–V5 stage (File S1).

Similarly, a small CO_2_ effect was observed in both genotypes. At V4–V5 and at R5–R6 stages respectively, 17 and 41 genes were differentially expressed in response to CO_2_ treatment (FDR < 0.05 and log_2_FC > 1) (File S1). The GSEA of all expressed genes revealed upregulation of sulphur metabolism, pentose and glucuronate interconversions and glycine, serine and threonine metabolism at the V4–V5 stage under e[CO_2_] (Figure 7C). The downregulated pathways under e[CO_2_] at the V4–V5 stage included isoflavonoid biosynthesis, photosynthesis, ribosome and porphyrin metabolism (Figure 7C). At the R6 stage, photosynthesis and porphyrin metabolism were still downregulated, and linoleic acid, alpha‐linolenic acid, plant‐pathogen interaction, glutathione metabolism, amino acid metabolism and glycolysis were upregulated under e[CO_2_] (Figure 7D).

Lastly, genes responsive to the interaction effect of line × CO_2_ were studied. At the V4–V5 stage, only 15 DEGs were significant for the interaction effect and clustered in four interaction clusters (File S1, Figure S12). At R5–R6 stage however, 283 genes were differentially expressed with top pathways being plant hormonal transduction, ribosome, spliceosome, RNA degradation, phagosome, oxidative phosphorylation and starch and sucrose metabolism as top pathways with genes greater than or equal to three. Genes significantly differentially expressed at the R5–R6 stage clustered into eight co‐expression clusters (File S1, Figure S13).

## 4 Discussion

Using a previously characterised transgenic line (VPZ‐34A), we set out to test the hypothesis that increases in light use efficiency, due to over‐expression of *AtVPZ*, and carbon assimilation from e[CO_2_], would have an additive effect on seed production in soybean, and to determine the molecular response to *AtVPZ overexpression*. However, we did not observe improvements in seed production or biomass in VPZ‐34A relative to WT, in ambient or e[CO_2_]. Instead, stem and seed biomass, along with total seed production per plot, were only influenced by [CO_2_] (Figure 5A–C). Importantly, despite seeing an increase in light use efficiency in VPZ‐34A, we didn't observe an increase in efficiency of carbon assimilation under fluctuating light, both in a[CO_2_] and e[CO_2_], which may underpin the lack of yield benefit, although further repetition will be necessary to draw firm conclusions.

### 4.1 VPZ‐34A Transcriptional Response to e[CO_2_] Broadly Similar to WT and Previous FACE Studies

In both VPZ‐34A and WT, the general transcriptional response to e[CO_2_] was consistent with previous studies. For example, upregulation of glycolysis at R5–R6 is consistent with previous transcript analysis at SoyFACE (Leakey, Xu, et al. 2009), as is a general downregulation of photosynthesis genes under e[CO_2_] (Leakey, Xu, et al. 2009; Bredow et al. 2025). Similarly, changes in transcript abundance of genes belonging to stress response and lipid metabolism pathways were consistent with data described in Ainsworth et al. (2006) and Bredow et al. (2025). Therefore, the transcriptional response to e[CO_2_] is broadly preserved in both VPZ‐34A and WT in the current study. Nonetheless, some of the responses to e[CO_2_] were different. For instance, we did not observe upregulation of protein degradation or upregulation of respiratory pathways during the vegetative stage as previously described by Ainsworth et al. (2006). These differences may be partly attributable to changes in environmental conditions between experiments, differences in techniques by which transcripts were evaluated (i.e. microarray vs. RNAseq), in the genotypes tested, and the time of day when samples were collected, as 21% of genes undergo circadian regulation (Locke et al. 2018).

### 4.2 Impact of e[CO_2_] on Relaxation of Photoprotection in WT and VPZ‐34A Lines

The relaxation times for the energy‐dependent (qE) and zeaxanthin accumulation‐dependent (qZ) NPQ components (τ_1_ and τ_2_) were significantly lower in VPZ‐34A than in WT, and remained unchanged across both [CO_2_] concentrations (Figure 1A,B). However, growth of WT under e[CO_2_] had a significant effect on both time constants, where τ_1_ decreased, and τ_2_ increased. Previously, it has been shown that thylakoid proton conductivity increases with higher CO_2_, leading to decreased NPQ and ∆pH by an unknown mechanism (Kanazawa and Kramer 2002; Kanazawa et al. 2017). However, this is unlikely to be the cause of altered kinetics of WT grown at e[CO_2_] because measurements were taken under ambient conditions and there was no effect on maximum NPQ (Figure 1C). Some insight may be provided by the increase in τ_2_ (qZ relaxation time); this relaxation phase is typically assumed to be related to the rate of conversion of Zeaxanthin to Violaxanthin by ZEP. The abundance of native *ZEP* transcripts trend downwards under e[CO_2_], with the decrease in *GmZEP1* significant (Figure S11). These findings are analogous to reports from Arabidopsis, and would be consistent with slower relaxation of qZ (Loladze et al. 2019). However, there was also a statistically significant decrease in *GmVDE1*, *GmVDE2*, *GmPsbS1* and *GmPsbS2* under e[CO_2_]. The magnitude of these changes at the protein level remains to be determined, and we did not assess post‐translational regulation, which has been shown to be important in regulating ZEP protein levels in response to environmental stresses such as high light and drought (Schwarz et al. 2015; Bethmann et al. 2019), but it is possible growth under e[CO_2_] causes a decrease in the ratio of ZEP to VDE protein accumulation, which could result in slower epoxidation of zeaxanthin and an increase in τ_2_. This interpretation would be consistent with an increase in Zeaxanthin content in plants grown at e[CO_2_], which may possibly occur (Figure 3J) but requires further validation as differences were not statistically significant. Taken together, these results reveal differential transcriptional regulation of *VDE* and *ZEP* in response to growth under e[CO_2_], but its potential effect on qZ relaxation is overridden in *VPZ* transgenics, meaning transgenic performance under fluctuating light will likely remain unaffected by future [CO_2_].

### 4.3 Potential Reasons for Lack of Increase in Seed Production in VPZ‐34A Lines Relative to WT

While the absence of an increase in seed production in VPZ‐34A contrasts with 1 year of findings from De Souza et al. (2022), it is consistent with the second year (taken during the same year analysed here). Sink limitation is commonly responsible for suppressing the benefits of increasing carbon assimilation (Ainsworth et al. 2002, 2011; Morgan et al. 2005; Bishop et al. 2015; C. Li et al. 2019), but it can be discounted as influencing the outcome, because we observed an increase in seed production in WT and VPZ‐34A under e[CO_2_] (Figure 5C). Rather, harvest index and 100‐seed weight were significantly impacted by VPZ expression, as previously seen for multiple VPZ events in soybean (De Souza et al. 2022). VPZ‐34A produced a greater number of pods and seeds (Figure 5E,F), but a reduction in 100‐seed weight offset any gains, resulting in a similar overall plot seed weight between lines under both ambient and e[CO_2_] (Figure 5C).

The observed effect of VPZ‐34A on transcription of native soybean genes during reproduction was small. Grain filling happens during the R5 stage, and by the R6 stage, seeds are already filled. Here, samples for transcriptomics were taken at an intermediate stage (R5–R6), which may have impacted our ability to fully detect transcriptional changes impacting reproduction. However, there we did identify transcriptional changes that may have impacted yield. Particularly, stage‐specific downregulation of key carbon metabolism genes such as *GAPC1* and *ENO2* in VPZ‐34A could have impacted the amount of photosynthate available for seed filling, which in turn, could be responsible for the smaller seeds in VPZ‐34A plants (Figures 6C,D, 7B). In addition, GSEA suggested pathway‐wide downregulation of carbon metabolism, but given the weak FCs, it cannot be confidently concluded that the changes are biologically meaningful. How AtVPZ affects carbon metabolism in a growth stage‐dependent manner, therefore, remains to be understood.

In addition, we found evidence for differential expression of two genes in VPZ lines with a potential role in seed development that could have influenced yield. *Glyma.09G248200* and *Glyma.18G244600* are homologues of Arabidopsis APETALA2 (AP2) domain‐containing protein, PLT4/BABY BOOM (*BBM*). *AtBBM* regulates embryo and endosperm development, and knockout mutants of *the AP2* gene are shown to have greater seed mass (Jofuku et al. 2005; B. Chen et al. 2022). The impact of lineage‐specific divergence of *AP2* transcription factors is not fully understood (Jiang et al. 2020; Kerstens et al. 2020), but upregulation of two putative developmental regulators as a result of *AtVPZ* expression, combined with our observation of smaller seeds in the VPZ‐34A line, warrants further investigation. Likewise, *FT2c*, which regulates flowering in soybean and its homologue in Arabidopsis, is known for leaf to shoot apex signalling to regulate flowering, was upregulated by VPZ‐34A at the V4–V5 stage, also pointing to altered sink development in transgenic plants (Jaeger and Wigge 2007; Wu et al. 2017). An important caveat to this analysis is that we only analysed transcriptional changes in leaves and not reproductive tissue, so it is unclear to what extent the observed transcriptional changes impact seed development. This may also be important as the transgenes themselves have the potential to impact seed development if expressed in reproductive tissues. For example, increased carotenoid content is correlated with decreased seed weight in chickpea and soybean (Abbo et al. 2005; Gebregziabher et al. 2022), and in Arabidopsis, ZEP was identified as the major‐effect locus for seed carotenoid content (Gonzalez‐Jorge et al. 2016). In addition, expressing *AtVPZ* in reproductive tissues could affect NPQ and rates of pod photosynthesis, which have been shown to contribute to 14% in soy seed weight, and up to 25% in wheat and barley (Bort et al. 1994; Maydup et al. 2010; Cho et al. 2023). Analysis of gene expression atlases suggests that there is significant expression of *AtGAPA*, *AtFBA* and *AtRBCS* in embryonic, pod and seed tissues in Arabidopsis, while a similar situation is true for homologous genes in soybean, indicating it is likely the transgenes are expressed to a significant level in reproductive tissues (Rhee 2003; Winter et al. 2007; Almeida‐Silva et al. 2023).

Taken together, it cannot be ruled out that transcriptional changes induced by VPZ over‐expression are impacting seed weight and pod number, through affecting carbon metabolism, developmental regulators or the transgenes themselves. It is therefore unclear whether impacts on seed weight can be decoupled from changes in leaf‐level photosynthesis, for example, by restricting transgene expression to leaves. Nonetheless, our data provide several testable hypotheses for future research.

### 4.4 Lack of Observable Increase in Efficiency of CO_2_ Assimilation in VPZ‐34A

VPZ‐34A was previously shown to possess increased theoretical quantum maximum light use efficiency (ΦPSII_max_) and increased theoretical quantum maximum efficiency of CO_2_ fixation (ΦCO_2, max_) under fluctuating light (De Souza et al. 2022). While we replicated the previously observed increase in ΦPSII_max_ in VPZ‐34A, we did not see a corresponding increase in ΦCO_2, max_ (Figure 2A,C). Furthermore, improvements in ΦPSII_max_ largely disappeared during reproductive stages (Figure 2D). This may potentially be attributable to the differential expression of the native *GmVPZ* genes, most notably an eight‐fold increase in of *Glyma.09G000600* between vegetative and reproductive stages, which is a ZEP‐like homologue responsible for native LxL cycling (Leonelli et al. 2024) and may offset any gains from introducing the *AtZEP* in transgenic lines (Figure S11). Daily measurements of photosynthesis and pigment contents of WT and VPZ‐34A in the field were broadly similar to those reported by De Souza et al. (2022), with increased NPQ and elevated Zeaxanthin contents during the middle of the day. These changes did not show statistical significance in this study (Figure 3), but the effect was clearer in De Souza et al. (2022). The discrepancy may in part be attributed to the environmental conditions, whereby in De Souza et al. (2022) peak irradiation reached ~2000 µmol m^−2^ s^−1^ at 2 PM, leading to the largest observable difference between WT and transgenic performance, whereas cloud cover at 2 PM obscured this response here (Figure 3A). This behaviour is also different from VPZ tobacco lines (Kromdijk et al. 2016), which showed similar maximum NPQ values and decreased Zeaxanthin content in mid to late afternoon relative to untransformed controls. In both tobacco and soybean, the lines and years with observed improvements in biomass correspond to improvements in ΦPSII_max_ and ΦCO_2, max_ (Kromdijk et al. 2016; De Souza et al. 2022). Without concurrent improvements in ΦPSII_max_ and ΦCO_2, max_, any gains in efficiency of light harvesting are not being used for CO_2_ fixation.

To explain why increasing ΦPSII_max_ may not lead to increased carbon assimilation, it is important to appreciate that ΦPSII_max_ is the product of two components: (1) *F*_*q*_ *'*/*F*_*v*_' and (2) *F*_*v*_ *'*/*F*_*m*_ *'* (Genty et al. 1989). Breaking down ΦPSII (*F*_q_ *'*/*F*_m_ *'*) at low light into these separate components reveals VPZ led to a decrease in NPQ in VPZ‐34A, as seen by an increase in *F*_*v*_ *'*/*F*_*m*_ *'*, without impacting photochemical quenching (Figure S5E–G). Thus, while there were more electrons available during vegetative stages, they were not used for photosynthesis.

These extra electrons were likely used in alternate electron sinks such as cyclic electron flow (CEF), respiration, photorespiration, nitrogen assimilation, malate shuttle or Mehler reaction. Walker et al. (2014) previously implicated the malate‐shuttle or Mehler reaction in balancing ATP/NADPH requirements under low light as opposed to CEF, and in accordance with these findings, we observed suppression of *Glyma.08G087100* in VPZ‐34A, a putative mitochondrial thioredoxin o1 (*Trxo1*), which would be consistent with alterations in redox metabolism. In Arabidopsis, Trxo1 is implicated in redox regulation of TCA cycle enzymes, alternative oxidase and photorespiratory enzyme subunit glycine‐decarboxylase (Reinholdt et al. 2019), and knockout lines had perturbations to photorespiration and the malate shuttle pathways (Fu and Walker 2023; von Bismarck et al. 2023). Our measurement conditions were unable to provide insight into whether photorespiration is perturbed in VPZ‐34A, but they provide an avenue for future research.

### 4.5 Altered Acclimation Response in VPZ‐34A to e[CO_2_]

Acclimation to e[CO_2_] has been shown to involve a reduction in content of both rubisco and light harvesting components, with rubisco typically lost to a greater extent, as photosynthetic limitation shifts towards maximum rates of electron transport and RuBP regeneration (Leakey, Ainsworth, et al. 2009). Consistent with this, e[CO_2_] grown WT leaves during vegetative stages showed a decrease in *V*_c, max_, *J*_max_ and a decrease in the ratio of *V*_c, max_:*J*_max_ (Table 1). Although VPZ‐34A also showed decreases in *V*_c, max_, *J*_max_ was largely unaffected by developmental stage or CO_2_ treatment (Table 1). Therefore, as with NPQ relaxation parameters, these findings may suggest WT plants adjust electron transport capacity in accordance with demand, whereas placing ZEP, VDE and PsbS under synthetic regulation abolishes this adjustment.

It is not obvious why there was no additive impact of e[CO_2_] on carbon assimilation of VPZ‐34A if *J*_max_ was higher and plants are not Rubisco‐limited, but our data suggest increased reductant was not being used for extra carbon fixation in the year measured. In addition, this study focused on sun‐exposed leaves at the top of the canopy, whereas assessment of whole canopy photosynthesis, along with transgenic and native VPZ protein contents during development, could help us understand the interplay between canopy architecture, whole plant physiology and photoprotection.

### 4.6 Reasons for Inconsistent Results of VPZ Transgenics Between Studies

The factors limiting photosynthetic efficiency will vary between species and environments, meaning it is likely that a synthetic *VPZ* genotype is only beneficial under some circumstances. Further, the number of paralogs of *VPZ* genes varies between species (Frommolt et al. 2008; Sulli et al. 2023), with the catalytic efficiency, expression profiles, substrate specificity and interaction behaviour between variants of *VDE*, *PsbS* and *ZEP* homologues largely unknown. The importance of this is highlighted by the unexpected stimulation of Lutein‐epoxide production in soybean transgenic lines expressing *AtZEP* (Figure S9A), a compound not known to accumulate in Arabidopsis, which has the potential to influence light harvesting efficiency and therefore overall *VPZ* transgenics performance (Esteban and García‐Plazaola 2014; Leonelli et al. 2024).

In addition to species‐specific differences, the lines reported in previous publications (Kromdijk et al. 2016; Garcia‐Molina and Leister 2020; De Souza et al. 2022; Lehretz et al. 2022) have varying degrees of transgene expression and physiological responses. This could be important for reasons distinct from photosynthetic efficiency under fluctuating light, for example, altered *VPZ* gene balance could result in excess NPQ, which would divert reductant from photosynthesis under high light and offset any potential gains (Zuo 2025). Elsewhere, Lehretz et al. (2022) observed decreased ΦPSII and CO_2_ assimilation under high light conditions, which may have contributed to the lack of observable change in performance. Conversely, simple increases in NPQ can also improve photosynthesis by reducing photodamage under high light (Hubbart et al. 2018), while faster induction of NPQ has also been proposed to be a contributing factor to improved performance by reducing photodamage resulting from energy imbalances during low‐to high light transitions (Long et al. 2022). Further, individual expression of *VDE* (Gao et al. 2010; Z. Chen and Gallie 2012; Yang et al. 2015; Sun et al. 2019), *PsbS* (Głowacka et al. 2018; Turc et al. 2024) or *ZEP* (Park et al. 2008; Zhang et al. 2016; Lou et al. 2017) has been shown to confer benefit in response to abiotic stresses plants may experience in the field. Taken together, the unresolved interplay between maximum NPQ, environmental interactions and plant physiology may further contribute to observed differences in *VPZ* response.

## 5 Conclusion

Our data show that the overexpression of *AtVPZ* genes in soybean consistently increases ΦPSII_max_, although this increase does not necessarily translate into increases in carbon assimilation and seed production. We show that growth at e[CO_2_] alters NPQ relaxation kinetics in WT soybean, and it is correlated with transcriptional downregulation of native *ZEP* genes, but this response is decoupled in *VPZ* plants where transgenes are driven by alternate promoters. Similarly, *VPZ* expression altered photosynthetic acclimation in response to e[CO_2_], as evidenced by a lack of decrease in *J*_*max*_. These findings may point to an inability to adapt photosynthetic capacity to demand in current *VPZ* transgenics. Gene expression analysis suggests secondary effects of *VPZ* expression, affecting photosynthate partitioning and seed development, potentially affecting the yield. Unravelling of the interplay and regulation of photosynthesis, photoprotection, sink capacity and development requires further investigation and will be key for realising the potential of *VPZ* expression as an engineering approach to increase crop yield.

## Supporting information

Supporting File 1

Supporting File 2

## Data Availability

Raw reads were deposited in the NCBI Sequence Read Archive (https://www.ncbi.nlm.nih.gov/sra), BioProject accession GSE270020.
