# Supplementary material for: Evaluating the Combined Effects of Elevated [CO2] and Accelerated NPQ Relaxation on Yield, Physiology and Transcription in Soybean
Source: Plant Cell Environ. 2026 May 11;49(8):6169–87. doi: 10.1111/pce.70608 (PMC13353578; doi:10.1111/pce.70608)
Supplement: Supplementary file 2 — Supporting File 2 [file PCE-49-6169-s001.docx]

**Supplementary Figures**

**Supplementary Figure S1.** Map of soil types (orange lines) for each experimental plot (red squares) and block (blue square) at the Soybean Free-Air CO_2_ Enrichment (SoyFACE) facility in Champaign, IL, USA with soil type legend and summary table by experimental plot.

**Supplementary Figure S2.** Daily averages of cumulative daily solar radiation **(A)**, cumulative daily precipitation **(B)**, daily maximum, minimum, and mean temperature **(C)**, daily maximum, minimum, and mean ambient CO_2_ **(D)**, and daily maximum, minimum, and mean vapor pressure deficit **(E)**.

**Supplementary Figure S3.** Light program for NPQ activation gas exchange protocol **(A).** Time points highlighted in red were used for calculations in Supplementary Figure S4. Non-photochemical quenching (NPQ) **(B-C)**, electron transport rate (ETR) **(D-E)**, and carbon assimilation **(F-G)** over time elapsed during vegetative (V4-V5) (**B, D, F**) and reproductive (R5-R6) **(C, E, G)** stages of WT and VPZ-34A grown at SoyFACE, Champaign, IL, USA under ambient and [CO_2_].

**Supplementary Figure S4.** Maximum quantum efficiency of CO_2_ assimilation (ΦCO_2, max_) **(A-B)**, maximum quantum efficiency of linear electron transport (ΦPSII_max_) **(C-D)**, and non-photochemical quenching (NPQ_lowlight_) **(E-F)** during NPQ activation from 50-200 PPFD (SF2-A) of WT and VPZ-34A grown at SoyFACE, Champaign, IL, USA under ambient and e[CO_2_] for vegetative (V4-V5) **(A, C, E)** and reproductive (R5-R6) **(B, D, F)** stages. Values represent repeated measurements from vegetative (V4-V5) and reproductive (R5-R6) stages. Significant differences between lines, [CO_2_] and interaction line*[CO_2_] are in bold and indicated by P▒<▒0.1 (*n*▒=▒4). Data presented in Supplementary Figure S3 at time points highlighted in red (Supplementary Figure S3A) was used to derive the values presented in this figure.

**Supplementary Figure S5.** Light program for fluctuating light gas exchange protocol **(A).** Time points highlighted in red were used for calculations in E-G. Photochemical quenching (qP) **(****B)**, Fv’/Fm’ **(C)**, and instantenous ΦPSII **(D)** over time elapsed during vegetative (V4-V5) stage. Means of qP **(****E)**, Fv’/Fm’ **(F)**, and instantaneous ΦPSII **(G)** from low light (▒<▒220 PPFD) after high to low light transitions of WT and VPZ-34A grown at SoyFACE, Champaign, IL, USA under ambient and **e**[CO_2_]. Values represent repeated measurements from vegetative (V4-V5) stage. Significant differences between lines, [CO_2_] and interaction line*[CO_2_] are in bold and indicated by P▒<▒0.1 (*n*▒=▒4).

**Supplementary Figure S6.** Light program for fluctuating light gas exchange protocol **(A).** Time points highlighted in red were used for calculations in E-F. Time points marked with red stripes were used for high light calculations (Supplementary Figure S8). Non-photochemical quenching (NPQ) **(B)**, electron transport rate (ETR) **(C)**, and carbon assimilation **(D)** over time elapsed during vegetative (V4-V5) stage. Mean linear model for slope calculations of theoretical maximum yield of photosystem II (ΦPSII_max_) **(E)** and theoretical maximum yield of carbon assimilation (ΦCO_2,max_) **(F)** calculated from 50-200 PPFD after high to low light transitions of WT and VPZ-34A grown at SoyFACE, Champaign, IL, USA under ambient and **e**[CO_2_]. Values represent repeated measurements from vegetative (V4-V5) stage. Significant differences between lines, [CO_2_] and interaction line*[CO_2_] for ΦPSII_max_ and ΦCO_2,max_ are shown in Figure [2](#MEP_L_fig2).

**Supplementary Figure S7.** Non-photochemical quenching (NPQ) **(A)**, electron transport rate (ETR) **(B)**, and carbon assimilation **(C)** over time elapsed during reproductive (R5-R6) stage under the fluctuating light (Supplementary Figure S6A). Mean linear model for slope calculations of theoretical maximum yield of photosystem II (ΦPSII_max_) **(E)** and theoretical maximum yield of carbon assimilation (ΦCO_2,max_) **(F)** calculated from 50-200 PPFD after high to low light transitions of WT and VPZ-34A grown at SoyFACE, Champaign, IL, USA under ambient and e[CO_2_]. Values represent repeated measurements from reproductive (R5-R6) stage. Significant differences between lines, [CO_2_] and interaction line*[CO_2_] for ΦPSII_max_ and ΦCO_2,max_ are shown in Figure [2](#MEP_L_fig2).

**Supplementary Figure S8.** Mean non-photochemical quenching (NPQ) **(A-B)**, electron transport rate (ETR) **(C-D)**, and carbon assimilation (A) **(E-F)** at high light (▒>▒1800 PPFD) in fluctuating light during vegetative (**A, C, E)** and reproductive (**B, D, F)** stages in WT and VPZ-34A grown at SoyFACE, Champaign, IL, USA under ambient and **e**[CO_2_]. Significant differences between lines, [CO_2_] and interaction line*[CO_2_] for ΦPSII_max_ and ΦCO_2,max_ are in bold and indicated by P▒<▒0.1 (*n*▒=▒4). For vegetative mean ETR and A at high light, the assumption of normality was not met. The non-parametric Kruskal-Wallis (KW) test was used to evaluate line and [CO_2_] effects independently. Data presented in Supplementary Figure S6-S7 at time points highlighted in red stripes (Supplementary Figure S6A) was used to derive the values presented in this figure.

**
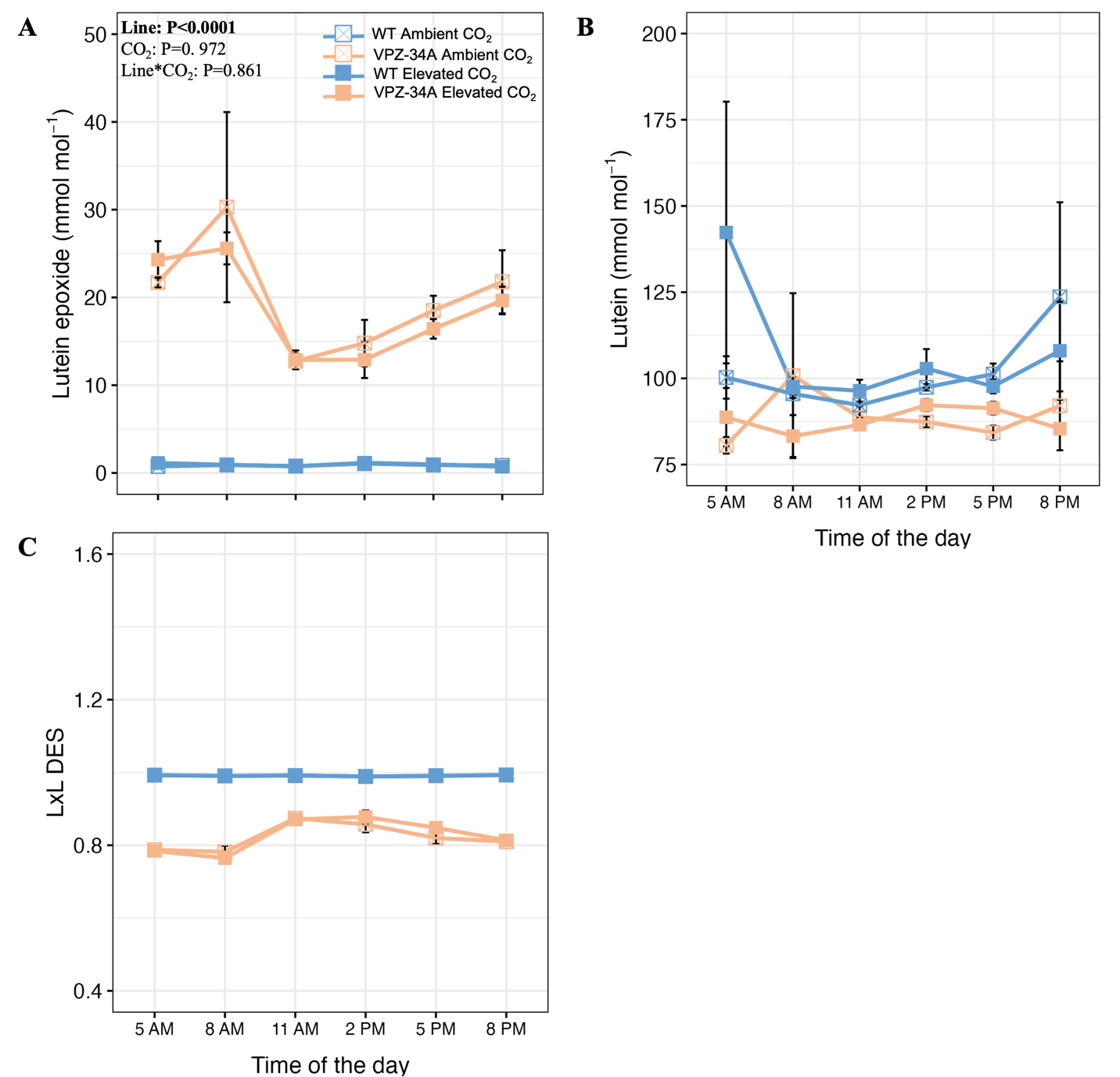
**

**Supplementary Figure S9.** Diurnal measurements of lutein epoxide (Lx) **(A)** and lutein (L) **(B)** levels as normalized by chlorophyll a content (mmol mol^-1^), and the de-epoxidation state of the lutein epoxide cycle (LxL DES) represented in percentage and calculated as L/(L+Lx) **(C)** across six timepoints in WT and VPZ-34A grown at SoyFACE, Champaign, IL, USA under ambient and elevated a[CO_2_]. Significant differences between lines, [CO_2_] and interaction line*[CO_2_] are in bold and indicated by P▒<▒0.1 (*n*▒=▒4). Lutein and LxL DES sample distribution failed to met the assumptions of gamma distributions. Removal of extreme outliers and data transformation didn’t provide sample distribution that can be modeled using gamma or normal distribution. Thus, p-values of LxL DES and Lutein were not reliable and are not provided.

|  | **Ambient CO_2_** | | | | | | | | **Elevated CO_2_** | | | | | | | |  |  |  |
| --- | --- | --- | --- | --- | --- | --- | --- | --- | --- | --- | --- | --- | --- | --- | --- | --- | --- | --- | --- |
| DAS | WT | | | | VPZ-34A | | | | WT | | | | VPZ-34A | | | |  |  |  |
| 8 |  |  |  |  |  |  |  |  |  |  |  |  |  |  |  |  |  |  | VE |
| 10 |  |  |  |  |  |  |  |  |  |  |  |  |  |  |  |  |  |  | VC |
| 16 |  |  |  |  |  |  |  |  |  |  |  |  |  |  |  |  |  |  | V1 |
| 17 |  |  |  |  |  |  |  |  |  |  |  |  |  |  |  |  |  |  | V2 |
| 25 |  |  |  |  |  |  |  |  |  |  |  |  |  |  |  |  |  |  | V3 |
| 30 |  |  |  |  |  |  |  |  |  |  |  |  |  |  |  |  |  |  | V4 |
| 35 |  |  |  |  |  |  |  |  |  |  |  |  |  |  |  |  |  |  | V5 |
| 38 |  |  |  |  |  |  |  |  |  |  |  |  |  |  |  |  |  |  | V6 |
| 44 |  |  |  |  |  |  |  |  |  |  |  |  |  |  |  |  |  |  | V7 |
| 45 |  |  |  |  |  |  |  |  |  |  |  |  |  |  |  |  |  |  | V8 |
| 51 |  |  |  |  |  |  |  |  |  |  |  |  |  |  |  |  |  |  | R1 |
| 59 |  |  |  |  |  |  |  |  |  |  |  |  |  |  |  |  |  |  | R2 |
| 65 |  |  |  |  |  |  |  |  |  |  |  |  |  |  |  |  |  |  | R3 |
| 74 |  |  |  |  |  |  |  |  |  |  |  |  |  |  |  |  |  |  | R4 |
| 81 |  |  |  |  |  |  |  |  |  |  |  |  |  |  |  |  |  |  | R5 |
| 87 |  |  |  |  |  |  |  |  |  |  |  |  |  |  |  |  |  |  | R6 |
| 111 |  |  |  |  |  |  |  |  |  |  |  |  |  |  |  |  |  |  | R7 |
| 126 |  |  |  |  |  |  |  |  |  |  |  |  |  |  |  |  |  |  | R8 |

**Supplementary** **Figure S10.** Developmental growth stages accessed from 8 days after sowing (DAS) to 126 DAS of wild-type (WT) and VPZ-34A grown at SoyFACE, Champaign, IL, USA under ambient and e[CO_2_]. Each column represents the data for one plot (*n*▒=▒4). VE = vegetative stage emergence; VC = vegetative stage cotyledon; V1-V8 = vegetative stage from the first fully developed trifoliate (V1) to eight nodes (V8); R1-R8 = reproductive stages.


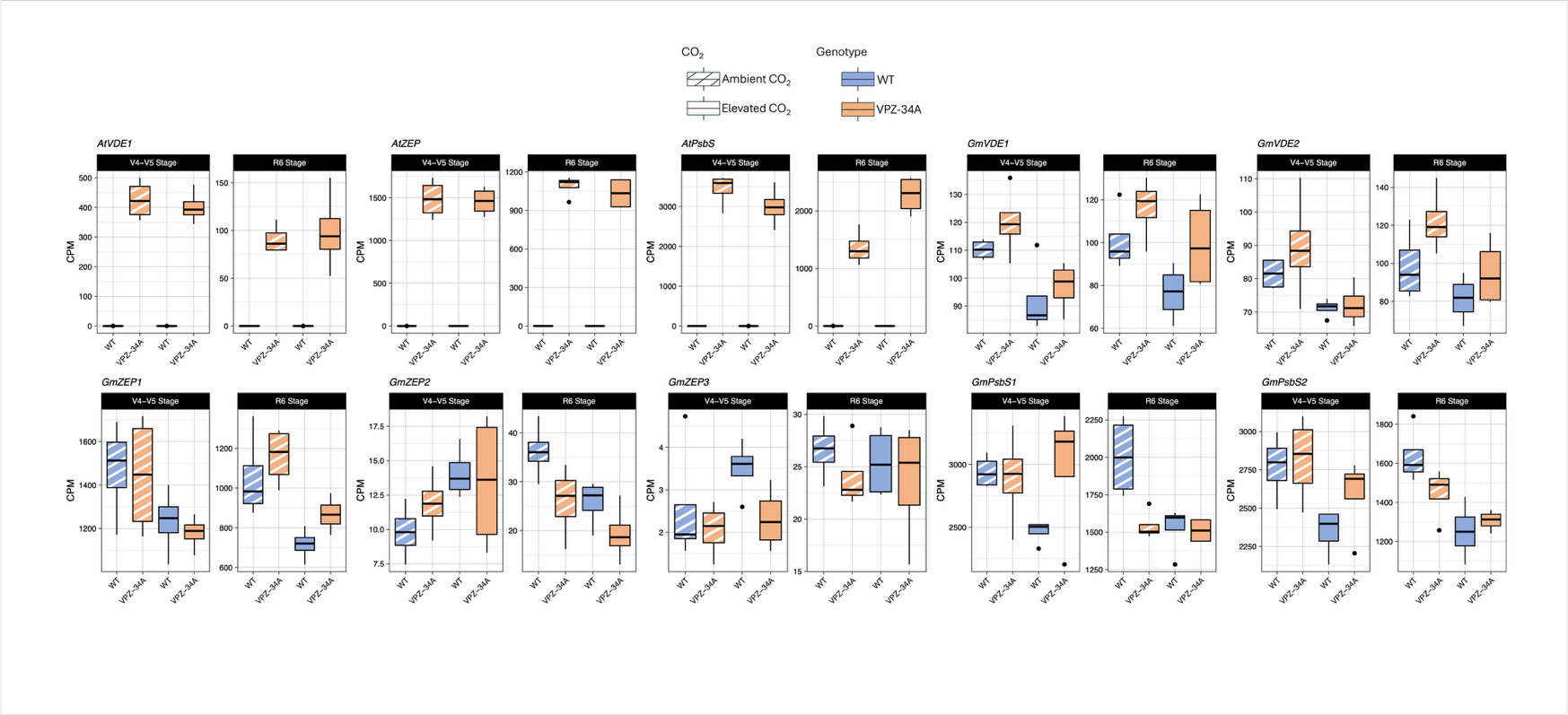


**Supplementary Figure S11.** Gene expression of three *AtVPZ* transgenes and native VPZ genes across all treatment x stage combinations.


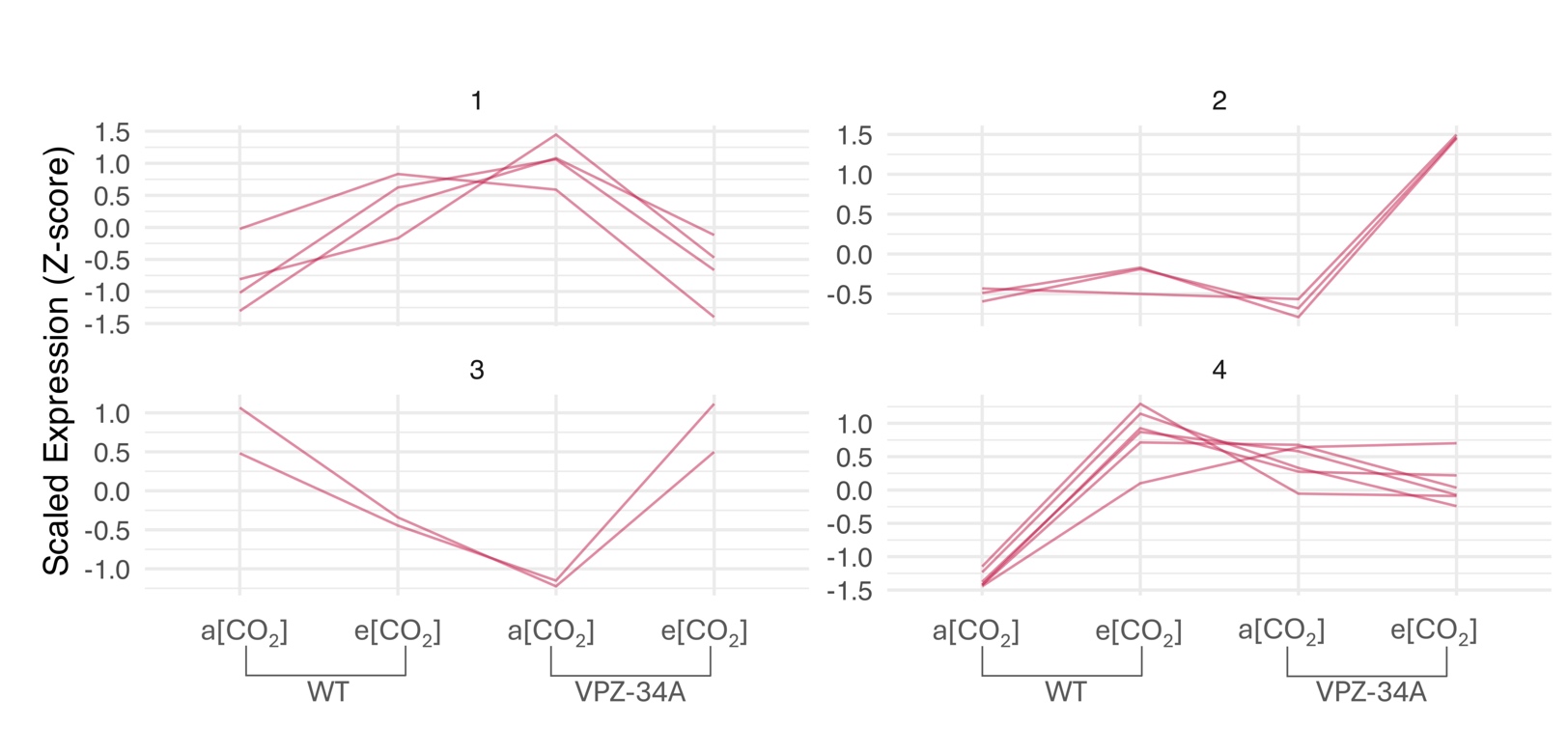


**Supplementary Figure S12.** Co-expression clusters among the line x CO_2_ responsive DEGs at V4-V5 stage. Co-expression clusters were created using using hierarchical clustering of the differentially expressed genes.


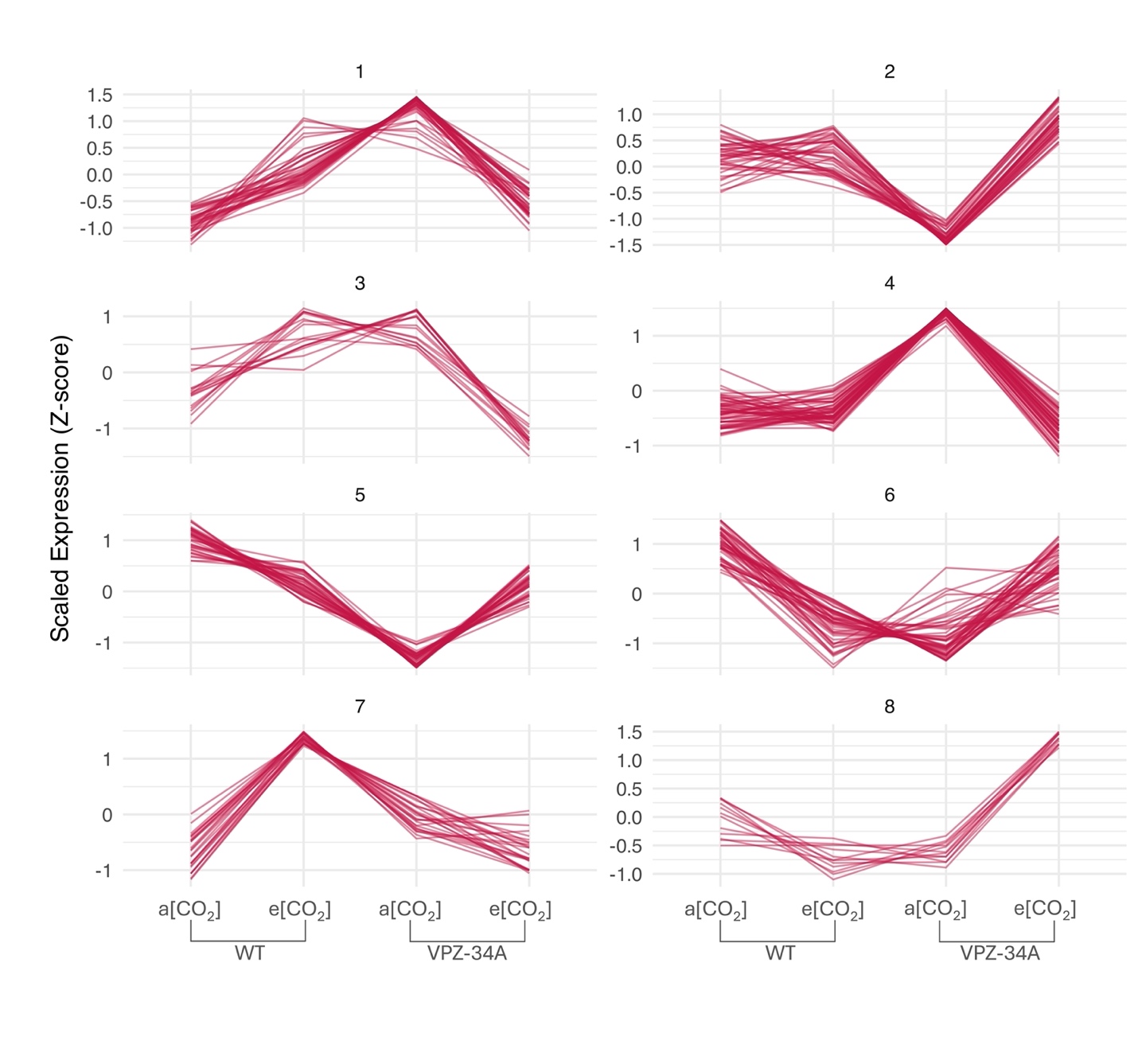
 **Supplementary Figure S13.** Co-expression clusters among the line x CO_2_ responsive DEGs at R6 stage. Co-expression clusters were created using using hierarchical clustering of the differentially expressed genes.
